# Characterization of the Oxidative Degradation Product of Darunavir by LC-MS/MS

**DOI:** 10.3797/scipharm.1505-10

**Published:** 2015-06-30

**Authors:** Karthik Yamjala, Jeevitha Atukuri, Krishnaveni Nagappan, Nivedeetha Halekote Shivaraju, Meyyanathan Subramania Nainar

**Affiliations:** Department of Pharmaceutical Analysis, JSS College of Pharmacy, [A Constituent College of JSS University, Mysore], Udhagamandalam, Tamilnadu-643001, India

**Keywords:** Characterization, Darunavir, Oxidative degradation, Validation

## Abstract

A rapid, selective, and reliable LC-MS^n^ method has been developed and validated for the isolation and structural characterization of the degradation product of darunavir (DRV). DRV, an HIV-1 protease inhibitor, was subjected to intrinsic oxidative stress conditions using 30% hydrogen peroxide and the degradation profile was studied. The oxidative degradation of DRV resulted in one degradation product. The unknown degradation product was separated on a Hibar Purospher C_18_ (250 mm × 4.6 mm; 5 µm) column by using 0.01 M ammonium formate (pH 3.0) and acetonitrile as mobile phase in the ratio of 50:50, v/v. The eluents were monitored at 263 nm using a UV detector. The isolated degradation product was characterized by UPLC-Q-TOF and its fragmentation pathway was proposed. The proposed structure of the degradation product was confirmed by HRMS analysis. The developed stability-indicating LC method was validated with respect to accuracy, precision, specificity/selectivity, and linearity. No prior reports were found in the literature about the oxidative degradation behavior of DRV.

## Introduction

The term “quality of pharmaceuticals” from the previous focus on purity is being changed to have a greater emphasis on impurities and degradation products. The use of pharmaceuticals is always a balance between risks and benefits, but the same is not true for impurities in pharmaceuticals; impurities carry only risks [1]. Stress testing has long been recognized as an important part in the drug development process. The focus on these degradation products and impurities is basically due to various health and safety considerations. A subset of these impurities may also cause potential genotoxicity, which poses an additional safety concern leading to carcinogenicity and genetic mutations.

Darunavir (DRV), (3*R*,3a*S*,6a*R*)-hexahydrofuro[2,3-*b*]furan-3-yl {(2*S*,3*R*)-4-[(4-aminobenzene-1-sulfonyl)(2-methylpropyl)amino]-3-hydroxy-1-phenylbutan-2-yl}carbamate is a potential drug used in the treatment of acquired immunodeficiency syndrome (AIDS) caused by human immunodeficiency virus [2]. It is the first line of drugs approved by the FDA to treat drug-resistant HIV [3]. It selectively inhibits the cleavage of HIV-encoded Gag-Pol polyproteins in virus-infected cells, thus preventing the formation of mature infectious virus particles [4].

Few analytical methods are available in the literature for the determination of DRV and other antiviral drugs in plasma by LC-UV [5, 6] and LC-MS [7–10]. Diastereomers and enantiomers of DRV were separated and quantified by LC and CE [11–13]. The quantification of DRV in different formulations was performed by HPTLC and LC [14–16]. Reddy *et al*. have reported a stability-indicating assay method for DRV [17]. Rao *et al*. reported an LC method for the quantification of DRV and its unknown impurities in bulk drugs [3], and also LC-MS/MS characterization of stress degradation products [18]. However, the oxidative degradation products of DRV were neither characterized nor were the fragmentation pathways proposed. To date, no study has been reported on the characterization of oxidative stress degradation products of DRV under ICH-prescribed stress conditions [19].

The aim of the present study was to understand the degradation behavior of the drug in an oxidative environment and to characterize the degradation products. It was achieved by exposing DRV to 30% hydrogen peroxide and analyzing the resultant solutions by optimized LC, high-resolution UPLC-MS/MS, and accurate mass measurements to elucidate the fragmentation pathway of DRV and its degradation product.

## Results and Discussion

### Optimization of Chromatographic Conditions

Initial separation conditions were tried on the Jones C_18_ column (150 × 4.6 mm id, 3 µm) and acetonitrile/water (50:50, v/v) was used as mobile phase for the separation of DRV and its degradation product. The chromatogram showed DRV and its degradation peak with tailing and poor resolution. To overcome these parameters, the mobile phase with different ratios such as 60:40, 65:35, 70:30, 75:25, and 80:20 were tried. These changes reduced the tailing but could not increase the resolution factor. Hence, to get acceptable resolution between DRV and its degradation peak, water was replaced with the buffer. Ammonium formate buffer (0.01 M, pH 3.0 adjusted with formic acid) /acetonitrile (50:50, v/v) in an isocratic mode and the Hibar Purospher C_18_ column (250 × 4.6 mm id, 5 µm) with stationary phase was used for the successful separation of DRV from its degradation product. The flow rate was 1.0 mL/min and the detection was carried out at 263 nm with 15 min of run time. These optimized conditions were used for the separation of DRV and its degradation product. For the LC-MS/MS and HRMS studies, the same method was used as HPLC, without the replacement of buffer.

### Degradation Behavior of DRV

**Fig. 1:**
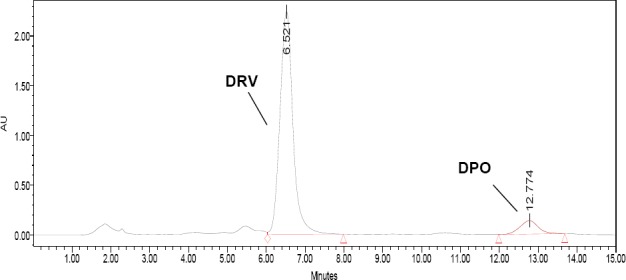
Typical HPLC chromatogram of DRV and DPO under oxidative degradation

DRV was subjected to oxidation using 3%, 6%, and 30% H_2_O_2_ for 15 days. It was observed that 3% and 6% H_2_O_2_ were ineffective at oxidizing the drug even after 15 days, whereas 30% H_2_O_2_ could degrade it after 15 days at room temperature. As a result, one degradation product (DPO) was formed. The HPLC chromatogram of the drug and its oxidative degradation product is given in Figure 1. The chromatographic and system suitability parameters such as retention time, relative retention time, peak purity, resolution, tailing factor, theoretical plates, and asymmetry factor for DRV and its degradation product were determined and are given in Table I.

**Tab. 1:**
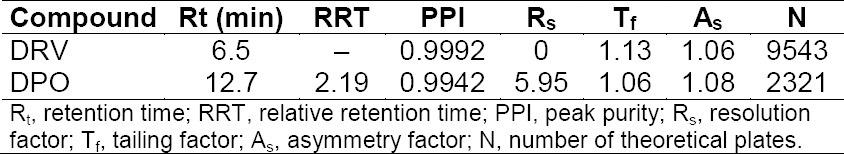
System suitability, peak purity and chromatographic data of DRV and DPO

### Isolation of DPO by Semi-Preparative HPLC

The target impurity (DPO) with a retention time of 12.7 min was separated by isocratic elution mode using the Zorbax RP C_18_ column (250 × 9.0 mm, id, 5 µm) and stationary phase with good resolution and a run time of 25 min. The isolated compound was re-confirmed by chromatographic analysis, the purity of DPO was found to be 99.8%, and this sample was used for mass analysis.

### Characterization Studies by LC-MS/MS

#### MS^n^ Study of DRV

**Fig. 2:**
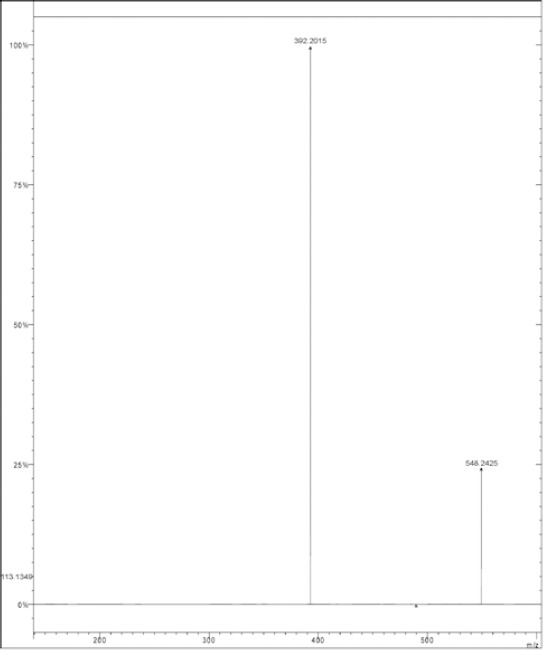
LC/MS spectra of darunavir

The MS^n^ spectral study of DRV and its fragmentation pathway was already reported in the literature and it was very well-supported by the HRMS data [18]. The MS spectra of DRV are reported in Figure 2.

#### MS^n^ Study of DPO

**Fig. 3:**
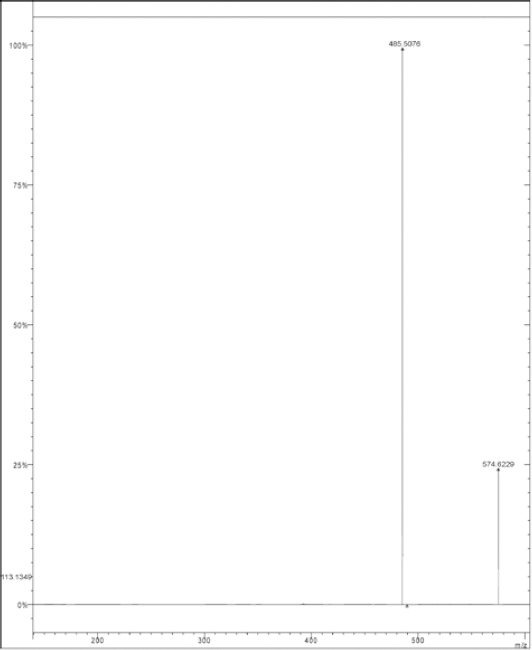
LC/MS spectra of DPO

**Fig. 4:**
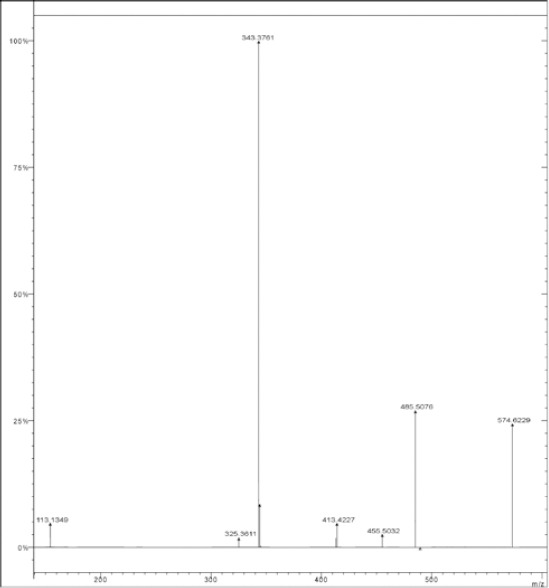
MS^n^ fragmentation spectra of DPO

**Fig. 5:**
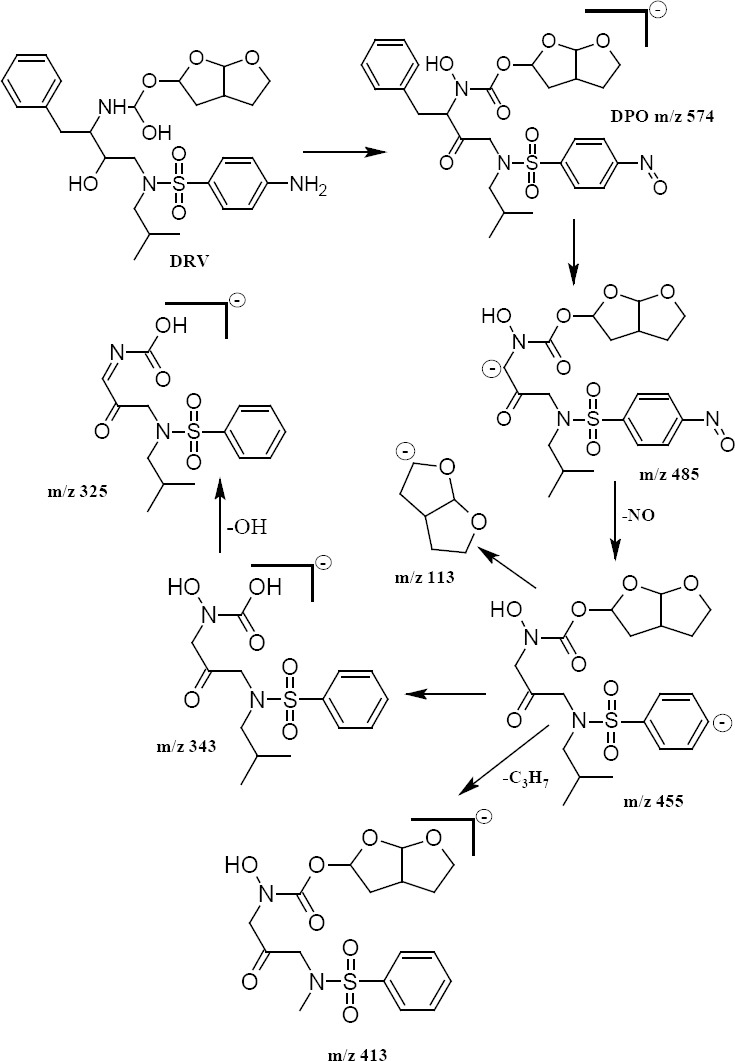
Plausible fragmentation pathway of the degradation product of DRV under oxidative stress conditions

The oxidation of DRV yielded one degradation product (DPO) which was formed in the presence of 30% H_2_O_2_ at room temperature for 15 days. The molecular ion peak of DPO appeared at m/z 574.634 Da (Figure 3). Its mass could be attributed to the formation of ketones at the 11- and 15-positions with the loss of one hydrogen atom and simultaneous oxidation at the 12- and 38-positions of DRV to form a hydroxyl amine and N-oxide, respectively. The MS^2^ fragment ions of DPO were at m/z 485, 455, 413, 343, and 325 (Figure 4). The elimination of a water molecule from m/z 343 yielded an m/z of 325, confirming the presence of a hydroxylamine group. The fragmented ion of m/z 343 was formed by the loss of the hexahydrofuro[2,3-*b*]furan moiety. Similarly, a peak at m/z 455 was contributed to the loss of N-oxide (m/z 30) from the fragment ion at m/z 485. The neutral ion (C_3_H_7_^-^) loss from m/z 455 resulted in the formation of a peak at m/z 413 (Figure 5). These results were further confirmed by HRMS data (Table II). The plausible structure of DPO and its fragmentation pattern are shown in Figure 5.

**Tab. 2:**
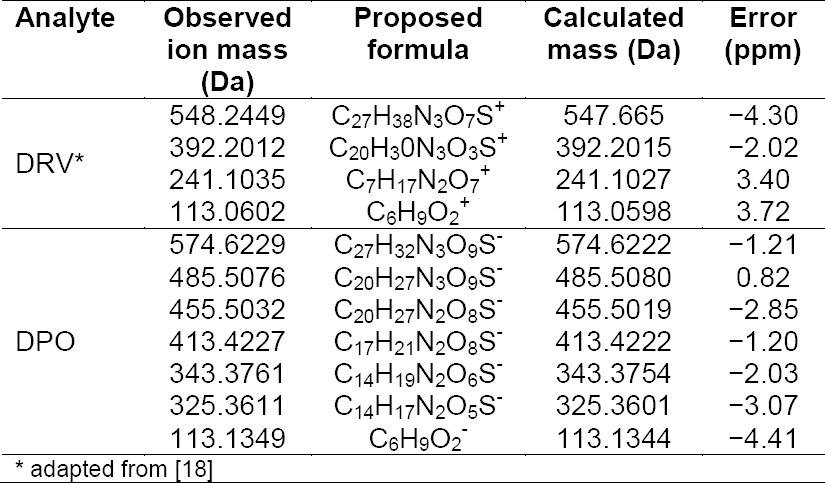
Elemental compositions of DRV and DPO in MS/MS spectra

### Method Validation

The developed LC method was validated as per ICH guidelines in terms of sensitivity, linearity, specificity, accuracy, and precision [20], and the data are summarized in Table III. The sensitivity of the analytical method was determined in terms of the LOD and LOQ which represent the concentration of analyte that would yield an S/N of 3 for the LOD and 10 for the LOQ. The detection and quantification limits of DRV were found to be 10 and 30 ng/mL, respectively. Excellent linearity was observed for DRV in the concentration range of 100-5000 ng/mL. The linear regression equation and correlation coefficient (r) were y = 37.326x + 9346.1, 0.9985, respectively. The specificity of the method was determined by injecting degradation solution of DRV. The drug was well-separated from the impurity and the method was found to be specific. The peak purity studies were performed on LC equipped with a PDA detector and were found to be 0.9992 and 0.9967 for DRV and DPO, respectively. The precision (intra- and interday) was assessed using three quality control samples. Six replicates were analyzed to determine the intraday precision. The procedure was repeated six times over three days in order to determine the interday precision. The accuracy of method was measured in terms of recovery. Three different concentrations were used and the recoveries of the drug were calculated from the difference between the peak areas of fortified and unfortified degraded samples. The effect of flow rate on the resolution was studied by altering the flow rate by ± 0.2 mL/min. The effect of pH on the resolution of DPO was studied by changing the pH by ± 0.1 units (buffer pH of 2.9 and 3.1). The resolutions between the closely eluting peaks were greater than 2.0, demonstrating the robustness of the method. The ruggedness of the method was checked by different analysts on different instruments (Shimadzu and Agilent HPLC). The critical parameters were assessed and found to be within the limits indicated, so the developed method was rugged enough for routine analysis.

**Tab. 3:**
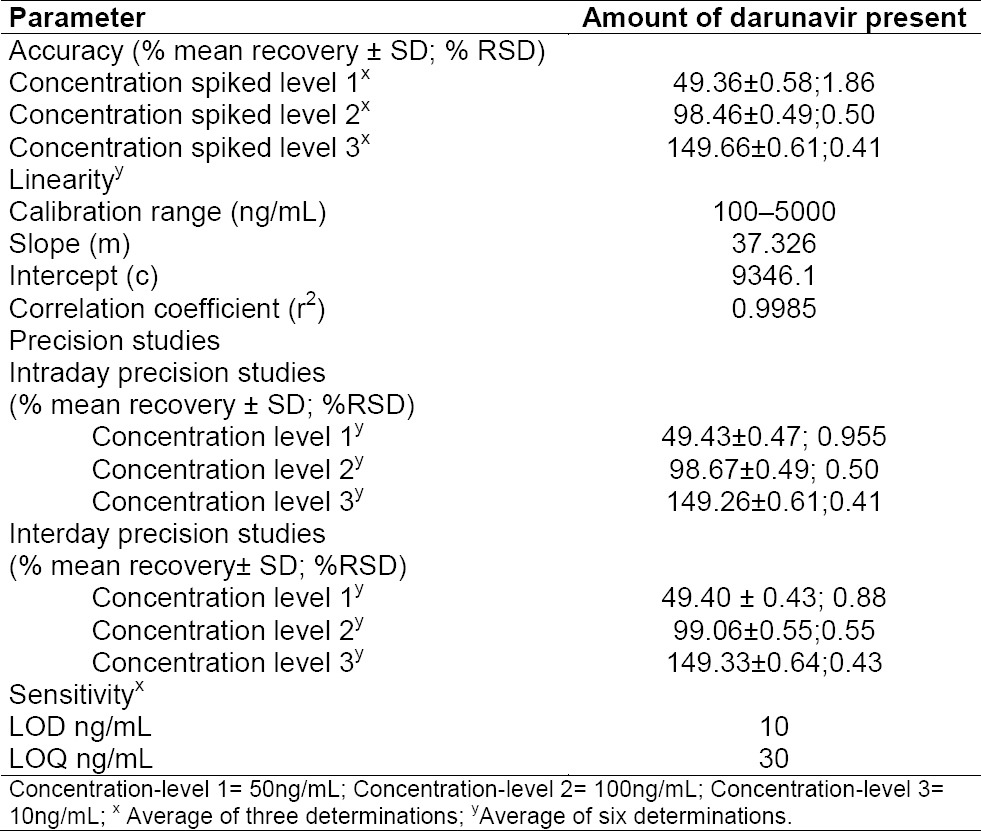
Validation summary

## Experimental

### Instrumentation and Reagents

#### Instrumentation

Degradation studies were investigated by using the Waters HPLC system equipped with a 1515 solvent delivery system, 2487 Dual Wavelength UV detector, and Rheodyne 7725*i* manual injector with 20 µl of loop volume. Peak purity and LC-MS experiments were carried out on the Shimadzu HPLC system equipped with an LC-20 AD pump, SPD-M20A PDA detector, and ESI-quadrapole mass spectrometer equipped with an SIL-20AC autosampler. A Hibar C_18_ column (250 × 4.6 mm, id, 5 µm) with stationary phase was used to separate all the compounds. The data acquisition and processing were performed using Lab Solutions software. LC-MS/MS studies and accurate mass measurements were carried out by the UHPLC quadrapole time-of-flight (Q-TOF) mass spectrometer (Agilent Technologies, USA) equipped with an ESI source. The data acquisition was under the control of Analyst^®^ QS software.

#### Chemicals

DRV (> 99% purity) was a gift sample from Mylan Pharmaceuticals, Hyderabad, India. HPLC grade acetonitrile (Merck, Mumbai, India) and HPLC water (Milli-Q Water Purification System, France) were used. Analytical reagent grade of ammonium formate, formic acid, and hydrogen peroxide were procured from S.D. Fine Chemicals (Mumbai, India).

### High-Performance Liquid Chromatography Conditions

A Hibar C_18_ column (250 × 4.6 mm, id, 5 µm) was used with mobile phase consisting of ammonium formate (pH 3.0, 0.01 M) and acetonitrile in an isocratic elution mode (50:50 %V/V) pumped through stationary phase at a flow rate of 1.0 mL/min at room temperature (16–20°C). The sample injection volume was 20 µL and eluents were monitored at 263 nm. Semi-preparative isolation of the DRV degradation product was carried out with the same mobile phase on the Zorbax C_18_ column (250 × 9.0 mm, id, 5 µm).

### Mass Spectrometry Conditions for MS/MS

All the samples (10 µL) were injected directly into the source by the flow injection method using ammonium formate (0.01 M, pH 3.0 adjusted with formic acid) and acetonitrile in the ratio 50:50 v/v as mobile phase at a flow rate of 0.5 mL/min. The mass spectra were recorded in ESI negative mode. Ultra-high purity nitrogen and helium were used as curtain and collision gas, respectively. The typical ion source conditions were: nebulizer gas, 60 psi; dry temperature, 325°C; dry gas, 5.0 mL/min; capillary voltage, 5kV; capillary current, 81.787 nA; vapourizer temperature, 400°C; dwell time, 200 ms. For the collision-induced dissociation (CID) experiments, the precursor ion was selected using the quadrapole analyzer and product ions were analyzed by the time-of-flight analyzer. HRMS data acquisition was performed by the following source conditions: capillary voltage, 5 kV; declustering potential (DP) and collision energy (CE) were −60 V and −10 V, respectively; focusing potential, 220 V; resolution 40,000 (FWHM).

### Sample Preparation and Forced Degradation Studies

Stock solution of DRV (5 mg/mL) was prepared by dissolving the appropriate amount in the diluent. The forced degradation studies of DRV were performed under oxidation conditions as per ICH guidelines [19]. The study was carried out with 3, 6, and 30% hydrogen peroxide at room temperature for 15 days and samples were withdrawn at regular time intervals and diluted ten times with the mobile phase and stored in the refrigerator at 4°C. Prior to HPLC and LC-MS/MS analysis, the sample solutions were filtered through a 0.45 µm membrane filter.

## Conclusion

The oxidative degradation behavior of darunavir (DRV) was studied as per ICH guidelines. One degradation product was formed during the study and was well-resolved from DRV by the proposed LC method. The proposed structure of DPO was characterized by MS^n^ analysis and was further confirmed by HR-MS data and accurate mass measurements. A simple, validated isocratic RP-LC method was developed for the determination of the stability-indicating assay of DRV and its degradation product.
